# Cerebral Vascular Toxicity after Developmental Exposure to Arsenic (As) and Lead (Pb) Mixtures

**DOI:** 10.3390/toxics12090624

**Published:** 2024-08-24

**Authors:** Keturah Kiper, Breeann Mild, Jenny Chen, Chongli Yuan, Ellen M. Wells, Wei Zheng, Jennifer L. Freeman

**Affiliations:** 1 School of Health Sciences, Purdue University, West Lafayette, IN 47907, USA; 2 Davidson School of Chemical Engineering, Purdue University, West Lafayette, IN 47907, USA

**Keywords:** arsenic, behavior, cerebral vasculature, lead, metal, neurotoxicity, zebrafish

## Abstract

Arsenic (As) and lead (Pb) are environmental pollutants found in common sites linked to similar adverse health effects. This study determined driving factors of neurotoxicity on the developing cerebral vasculature with As and Pb mixture exposures. Cerebral vascular toxicity was evaluated at mixture concentrations of As and Pb representing human exposures levels (10 or 100 parts per billion; ppb; µg/L) in developing zebrafish by assessing behavior, morphology, and gene expression. In the visual motor response assay, hyperactivity was observed in all three outcomes in dark phases in larvae with exposure (1–120 h post fertilization, hpf) to 10 ppb As, 10 ppb Pb, or 10 ppb mix treatment. Time spent moving exhibited hyperactivity in dark phases for 100 ppb As and 100 ppb mix treatment groups only. A decreased brain length and ratio of brain length to total length in the 10 ppb mix group was measured with no alterations in other treatment groups or other endpoints (i.e., total larval length, head length, or head width). Alternatively, measurements of cerebral vasculature in the midbrain and cerebellum uncovered decreased total vascularization at 72 hpf in all treatment groups in the mesencephalon and in all treatment groups, except the 100 ppb Pb and 10 ppb As groups, in the cerebellum. In addition, decreased sprouting and branching occurred in the mesencephalon, while only decreased branching was measured in the cerebellum. The 10 ppb Pb group showed several cerebral vasculature modifications that were aligned with a specific gene expression alteration pattern different from other treatment groups. Additionally, the 100 ppb As group drove gene alterations, along with several other endpoints, for changes observed in the 100 ppb mix treatment group. Perturbations assessed in this study displayed non-linear concentration-responses, which are important to consider in environmental health outcomes for As and Pb neurotoxicity.

## 1. Introduction

The cerebral vascular system in the brain plays vital roles in nutrient delivery, waste removal, and ensuring continuous blood perfusion, crucially forming during embryogenesis. Developmental defects in this network can severely impair brain function by reducing nutrient supply, hindering waste removal, and compromising the integrity of brain endothelial cells (BECs), which line the brain’s blood vessels with tight junction (TJ) proteins. A few environmental toxicants are reported to interfere with the development of the cerebral vascular system [1,2,3,4,5,6,7,8,9,10,11,12], but more studies are needed to further understand risks for toxicants that are found at common sites such as metalloid arsenic (As) and heavy metal lead (Pb).

As exists in organic and inorganic forms, with inorganic arsenic being more toxic and widely present in natural and human-influenced environments. Arsenate (As^5+^) and arsenite (As^3+^) are common forms of inorganic arsenic that, when metabolized in significant quantities, can induce toxicity. Metabolism of these forms generates reactive nitrogen species (RNS) and reactive oxygen species (ROS), which in excess can cause oxidative stress. These metabolites of As produced during detoxification are more toxic than their parent compounds with excess of RNS, ROS, methylarsonous acid (MMA^3+^), and dimethylarsinic acid (DMA^5+^) further inducing toxicity [13]. While the US Environmental Protection Agency (US EPA) concluded that As is a human carcinogen, other diseases are also implicated with As exposure, including neurotoxicity. For example, the US EPA reference dose for inorganic As is 0.0003 mg/kg/day based on effects on the skin (hyperpigmentation and keratosis) and possible vascular effects reported in epidemiologic studies from As exposure via contaminated drinking water [13]. As exposure across the globe occurs from naturally high concentrations of inorganic As occurring in geologic stores in some countries contaminating drinking water sources and also from industrial sources that can also contaminate water and food. Regulatory agencies developed guidance and limits for ingestion of water and food contaminated with As. For example, the US EPA’s maximum contaminant level (MCL) is 10 parts per billion (ppb, µg/L) in drinking water.

Similar to inorganic As, Pb is also found throughout the globe, but with only a small percentage of current Pb from a natural source. Most Pb in the environment is from historical and current industrial processes and past use as a gasoline additive [14]. Pb^2+^ salts and organic Pb compounds are listed as the most harmful. Pb in its Pb2+ oxidative state imitates calcium (Ca^2+^) and can travel via the bloodstream to wreak havoc on sensitive targets such as the brain. Research indicates that Pb exposure is not safe at any dose or duration and is especially dangerous for children [14]. The World Health Organization (WHO) recommends that children with blood Pb concentrations ≥ 5 µg/dL should identify the source of Pb exposure and take action to eliminate the exposure. Recently, the US Centers for Disease Control and Prevention (US CDC) lowered the blood Pb reference value from 5 µg/dL to 3.5 µg/dL in the blood of children [14]. This was an additional decrease from the change enacted in 2012 that lowered the blood Pb action level from 10 µg/dL to a blood Pb reference value of 5 µg/dL. Pb exposure primarily occurs via ingestion of contaminated drinking water from pipes and contaminated dust or chips from paint. Similar to As, global agencies provide guidance and have set regulatory limits to work to prevent and reduce Pb exposure. For example, the US EPA’s action level for Pb in drinking water is 15 ppb, while the US CDC recommends action if levels exceed 5 ppb to protect public health.

As and Pb are developmental toxicants. As is reported to perturb several structures in the central nervous system, including neurons and astrocytes [15]. Alterations in synaptic formation and suppressed protein expression of Ca^2+^ at N-methyl-d-aspartate receptors (NR1, NR2A, and NR2B), calmodulin (CaM)-dependent protein kinase II (CaMKII), and adenylate cyclase (AC) in neurons after 72 h of exposure to 0, 5, or 10 µM As^3+^ in the adult mouse brain were observed. The levels of Ca^2+^/CaM are signals to activate a functional protein in neurons that control activation of other synaptic targets that facilitate synaptic transmission and plasticity. As exposure is also reported to alter astrocytes, which changes neurochemistry associated with memory [16,17]. Developmental As exposure from gestation to lactation to 0, 0.15, or 15 mg/L As_2_O_3_ in drinking water resulted in a significant decrease in mRNA expression of TJ proteins (occludin, claudin, ZO-1, and ZO-2) and occludin protein expression in mice [18]. 

Pb is well established as a developmental neurotoxicant with long-term low dose exposure resulting in neurological functional changes throughout the lifespan of humans and in multiple animal models [19,20]. Pb exposure leads to its deposition in bones, a process influenced by age-related absorption kinetics. In adults, Pb accumulates in cortical and trabecular bones, readily releasing into the bloodstream. This stored Pb poses ongoing internal exposure risks, particularly concerning pregnant women and fetal development. Pb circulating in the blood can cross the placental blood barrier [21,22], the cerebrospinal fluid barrier [23], and the brain–blood barrier (BBB) [24,25,26,27], exposing offspring at all stages of in utero development. This exposure can result in serious effects to the developing fetus and infant, including damage to the baby’s central nervous system. In animal studies, Pb exposure via drinking water at postnatal day (PND) 20–22 in rats for 8 weeks resulted in severe leakage of the BBB and significant decreases in the TJ protein occludin and ZO-1. In addition, other studies also demonstrate the presence and accumulation of Pb in BECs and neurons, which in combination with decreased permeability as the BBB develops, highlights the increased risk of Pb exposure during development.

As and Pb exposure is also related to cardiovascular toxicity [28,29,30,31,32]. Chronic oral inorganic As exposure is associated with vascular lesions with in vitro studies, suggesting endothelial cell toxicity as a result of endoplasmic reticulum (ER) stress. The culprit of ER stress is proposed to be mediated by ROS signaling along with other protein signaling [33]. For Pb, an epidemiology study found that exposure at levels considered to be safe was significantly related to an increased risk of anemia through inhibited production of hemoglobin [34]. Pre- and post-natal exposure to Pb leads to abnormal morphology in wild-type and cerebral cavernous malformations 3 (*CCM3*) knockout models. *CCM3* is another essential gene that assists in cell proliferation, differentiation, and apoptosis during cardiovascular development. Furthermore, Pb exposure in primary human and mouse umbilical vein endothelial cells resulted in increased mRNA expression of hypoxia-inducible factor 1-alpha (*HIF-1α*), a regulator of mitochondrial metabolism and mediator of ischemic preconditioning. This change in *HIF-1α* was supported by an increase in transcription factor A mitochondrial (*TFAM*) [7], which indicates that inappropriate vascular development could occur given Pb exposure alone and in the presence of damaged *CCM3*. In adult rats exposed to low-to-moderate Pb decreased integrity of vascular endothelial cells, increased blood–retina barrier (BRB) permeability, and decreased expression of BRB claudin-5 was observed [35]. As such, combining the observations of As and Pb exposure on the central nervous and cardiovascular systems, there is a need to further investigate perturbations on the cerebral vascular system. In addition, given the presence of As and Pb at common environmental contamination sites, it is important to understand both single chemical and mixture exposures. 

The zebrafish (*Danio rerio*) is an established biological animal model and is an excellent candidate for developmental toxicity studies given several strengths. Transparency of the chorion allows visualization of the embryo in real time at the earliest developmental stages. In addition, high gene function similarity to mammals, a sequenced genome, and a large database of comprehensive transcriptome data enables swift detection of mechanisms underlying a toxicant’s effect and translation of findings to humans [36,37]. Specifically, identification of important markers in vascular endothelial cells and red blood cells in the zebrafish quickly became useful to analyze the development of the cardiovascular system [38]. The zebrafish brain also shares similarities to mammals [39]. In zebrafish, the blood–brain barrier (BBB) is composed of capillary endothelial cells delivering blood to brain tissue. These endothelial cells are connected by tight junctions and interface with pericytes. Zebrafish also possess radial glial cells akin to mammalian astrocytes. These radial glial cells contain blood vessels and act as neural stem cells and scaffolding cells for the development and migration of new neurons forming cortical layers [40]. Moreover, the development and function of the zebrafish BBB is well detailed [41,42,43,44]. 

Zebrafish are also being used to better understand mechanisms and outcomes of acute and chronic exposure to As or Pb. For example, chronic low-dose As^3+^ exposure in developing zebrafish was linked to changes in morphology and size, neural development, cell proliferation, apoptosis, and locomotor responses [45,46,47,48,49]. In addition, embryonic exposure to 100 ppb (µg/L) Pb results in significant changes in the expression of genes associated with neurological development and disease as well as altered protein expression and decreased axonal density [43,50]. Furthermore, other studies show that 10 and 50 ppb Pb treatments during embryogenesis alters expression of genes associated with the gamma-aminobutyric acid (GABA)-ergic system [51] and that embryonic Pb exposure ranging from 620 to 2000 ppb leads to behavioral alterations in adult zebrafish [52]. Overall, these studies support the use of the zebrafish to investigate developmental cerebral vascular toxicity of As and Pb. Thus, the purpose and novelty of this study was to determine the potential effects of a joint interaction of As and Pb in a mixture in the developing zebrafish, specific to cerebral angiogenesis and vasculogenesis, using wild-type zebrafish and a transgenic model in which the vascular system fluoresces [Tg(fli1:EGFP)].

## 2. Materials and Methods

### 2.1. Animal Husbandry

The 5D wild-type strain (behavior, light microscopy, and qPCR) or the Tg(fli1:EGFP) transgenic zebrafish line (confocal microscopy; obtained from the Zebrafish International Resource Center) were used in this study. Adult zebrafish were maintained at 28 °C under a light/dark cycle ratio of 14:10 hrs and kept in an open flow system with pH between 7.0 and 7.3 and salinity at 550 µS/cm conductivity. The adult zebrafish were maintained, fed, and bred according to established protocols [43,53]. Embryos were obtained by placing adults in spawning tanks with embryo collection occurring immediately after fertilization. This step was completed with ease as zebrafish have a photoperiodic nature with mating triggered by sunrise or natural lighting in a laboratory setting. Embryos used in this study were collected between the 2 and 16 cell stage and treated with chemical solutions listed in Section 2.2 for further experimental procedures through 72 or 120 h post fertilization (hpf). All protocols were approved by the Purdue University Institutional Animal Care and Use Committee (PACUC) with all fish treated humanely and with regard to the alleviation of suffering. 

### 2.2. Arsenic (As) and Lead (Pb) Sub-Lethal Mixture Chemical Exposure

The concentrations used for all assays performed in this study were several magnitudes below those that result in lethality [54]. In addition, concentrations reflect those found in drinking water and/or other environmental samples [e.g., the regulatory limit set by the World Health Organization and the US EPA for As in drinking water at 10 ppb (µg/L)]. As such, treatment concentrations were: 0 ppb (negative control, water only), 10 or 100 ppb As or Pb single chemical exposures, and mixtures consisting of 10 ppb As and 10 ppb Pb or 100 ppb As and 100 ppb Pb. Exposures were initiated immediately after fertilization (1 hpf) through 72 or 120 hpf. Developing zebrafish were immersed in 20 mL of the exposure solutions in groups of 50 (considered subsamples) within a Petri dish. Biological replicates were blocked by clutch with the embryos within a single Petri dish considered as subsamples of one biological replicate. Embryo collection and treatment with the above-mentioned solutions occurred several times to attain multiple biological replicates. Concentrations of dosing solutions were confirmed to be within expected concentrations using US Environmental Protection Agency-approved water test kits (As: Industrial Test Systems; Pb: Osumex).

### 2.3. Visual Motor Response Assay 

Larval behavior was assessed using a visual motor response (VMR) test at 120 hpf (chemical exposure from 1 to 120 hpf) to evaluate if developmental exposures altered behavior. At 120 hpf, zebrafish larvae exhibit defined locomotion patterns given rearing is consistent and standard [55]. Twelve larvae from each treatment (subsamples from within a single Petri dish for each treatment group) were placed into individual wells of a 96-square well plate to consist of one biological replicate. Each well was filled with 0.5 mL of embryo water. The treatments were loaded into 96-square well plates in a balanced order between columns to reduce any potential location-based artifacts. Locomotor behavior of the larvae was in response to a visual stimulus, white light, and was assessed using a DanioVision observation chamber and EthoVison XT video tracking software (Noldus, Leesburg, VA, USA). An infrared camera traced the larval movement and recorded at a rate of 25 frames per second. A 10-min acclimation period inside the Noldus DanioVision observation chamber was included. The white light routine included five alternating cycles of three cycles of dark light (10 min each) and two cycles of white light (10 min each), totaling 50 min (i.e., dark, light, dark, light, and dark). During the light phase, a 5000 lx light was activated under the DanioVision observation chamber. The infrared movement traces were recorded at a rate of 25 fps with a Basler GenICam acA1300–60 gm camera (Basler, Exton, PA, USA). Tracks were smooth via a minimum distance moved profile set to >0.2 mm. Parameters measured included distance traveled, velocity, and time spent moving during each phase. Temperature was held constant at 28 °C during the assay. All neurobehavioral tests were performed between 11am and 1pm to eliminate influence from circadian rhythm. The EthoVision software was used to smooth tracks via a minimum distance moved profile set to >0.2 mm. A total of six biological replicates were completed, each with 12 subsamples for each treatment group. 

### 2.4. Measurements of Larval Length, Head Length and Width, and Brain Length

Developing zebrafish were exposed to treatments from 1 to 120 hpf and at 120 hpf developmental morphological assessments were completed using light microscopy. Total body length (distance from snout to tail), head length (distance from snout to operculum), head width (distance of intraocular space from midpoint of each eye), and brain length (distance from rostral aspect of forebrain to caudal aspect of brainstem) were measured using a Nikon SMZ1500 dissecting microscope with NIS Element imaging software as described (Nikon, Boston, MA, USA, version 5.21) [56]. Total body length, head length, and head width were measured on dorsal view and brain length was measured on lateral view. In addition, ratios of head length, head width, and brain length to total larval length were calculated. Ten subsample larvae (from one Petri dish) were measured per biological replicate. Seven biological replicates were assessed. 

### 2.5. Confocal Microscopy Measurements of Cerebral Vascular Development

The motivation to observe the cerebral vasculature was determined by an earlier study evaluating acute lethal mixture toxicity of As and Pb in which micro-bleeding was observed in the brain of developing zebrafish at the LC_25_ of Pb and the lowest mixture group (Pb LC_25_ + As LC_25_) (Figure S1) [54]. Whether Pb and As single and mixture exposures at sub-lethal concentrations would disrupt development of the cerebral vasculature system was determined. Developing zebrafish were exposed to chemical treatments from 1 to 72 hpf and confocal measurements completed at 72 hpf. Larvae were anesthetized using diluted tricaine solution. The larvae were mounted onto a cover slide with a low melt agar solution and positioned from superior to inferior axis. A 10x objective air lens on a Nikon Intravital MP upright confocal equipped with a hybrid scanner for both high resolution and high-speed applications with higher resolution for open-face confocal imaging was used with NIS-Elements imaging software (Nikon, Boston, MA, USA, version 5.21) for the 2D and volume view and confocal z-stacks converted to a 3D image. Six specific endpoints were assessed: (1) the mean of the length of the mesencephalon (midbrain) and cerebellum (hindbrain), (2) the number of sprouting pre-tip blood vessels in the mesencephalon and cerebellum, (3) the number of connecting arteries in the mesencephalon and cerebellum (branches), (4) the mean total vasculature (as number of branches and sprouts), (5) the ratio of sprouting to branching, and (6) the ratio of blood vessels (branches) to length of mesencephalon or cerebellum were measured. Measurements were scored two different times with an evaluator blind to treatments. Example images from the mesencephalon (midbrain), cerebellum (hindbrain), and total brain of the fli1:1a transgenic fish are shown in Figure S2 and additional method details in Supplementary Methods. 

### 2.6. Quantitative PCR to Evaluate Gene Expression

Zebrafish embryos were exposed from 1 to 72 hpf in groups of 50 embryos per petri dish to 0 ppb, 10 ppb As or Pb, 100 ppb As or Pb, a mix of 10 ppb As and Pb, or a mix of 100 ppb As and Pb. At 72 hpf, fish from a single Petri dish were pooled and homogenized in Trizol. A total of 6 biological replicates (n = 6) were collected. RNA was isolated using the RNEasy Mini Kit (Qiagen, Germantown, MD, USA) and cDNA synthesized using the SuperScript IV First-Strand Synthesis System (Invitrogen, Waltham, MA, USA) with established protocols [57]. 

Genes identified as essential in development of vasculature and TJs were identified and examined for changes in expression resulting from exposure to individual chemicals and in mixture treatments. Gene targets included *cldn5a*, *cldn5b*, *lrp1aa*, *vegfaa*, and *wnt7aa* and expression was normalized to beta-actin (*actb2*) following similar methods as described in and implementation of MIQE guidelines [50,58,59,60,61]. Beta-actin was included as the reference gene given consistent expression that did not change with the chemical treatments. Forward and reverse primer pairs for target genes were generated using Primer3 [62] (Table S1) and checked through in silico analysis using NCBI Primer-BLAST (version 2.5.0). qPCR analysis was performed using BioRad SSoAdvanced Universal SYBR Green Supermix (Biorad, Hercules, CA, USA) on a CFX Connect Real-Time PCR Detection System (Biorad, Hercules, CA, USA) following manufacturer protocol [50]. The cycling parameters included a 3 min incubation phase at 95 °C followed by 40 cycles of 95 °C for 10 s, 60 °C for 30 s, and 72 °C for 30 s. Efficiency (100 + 10%) and specificity were confirmed with dissociation curves, non-template amplification controls, and a standard curve. All experimental samples were run in triplicate to provide technical replicates with all seven test concentrations included on a single 96-well plate. As such, 6 plates were included for each target gene of interest (6 biological replicates). 

### 2.7. Statistical Analysis

A Grubbs statistical test by the treatment group was run to identify any outliers and data confirmed to meet ANOVA requirements. Statistical analysis for the visual motor response assay was assessed for each measurement (i.e., distance traveled, velocity, and time spent moving) with repeated measures ANOVA using SAS. F statistics, *p*-values, and partial eta-squared value are reported for the interactions of each outcome. Light and confocal microscopy measurements and qPCR results were statistically analyzed with a one-way ANOVA and a least significant difference post-hoc test when significance was observed using SAS. Statistical significance was set at an α = 0.05. 

## 3. Results

### 3.1. Larval Behavior

**Figure 1:**
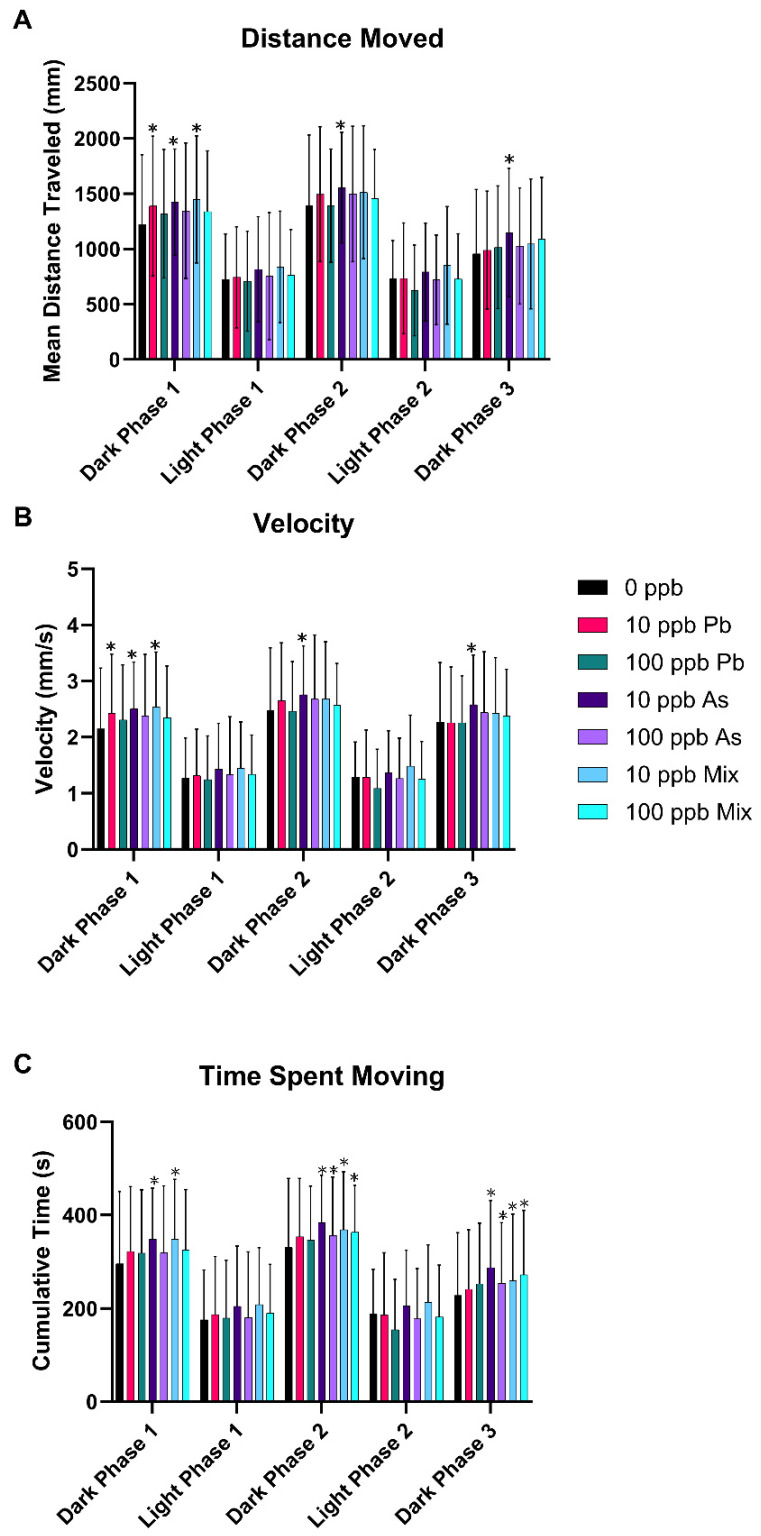
Neurobehavioral endpoints after developmental exposure to As, Pb, or a mixture of both from 1 to 120 hpf. Zebrafish larvae behavior was measured using the visual motor response assay and distance traveled (**A**), velocity (**B**), and time spent moving (**C**) measured among the dark and light phases. n = 6 with 12 subsamples per treatment per replicate to total 72 fish per treatment group. Error bars represent standard deviation. * *p* < 0.05.

Zebrafish were exposed to either 10 or 100 ppb As, Pb, or a mixture of As + Pb and behavioral response recorded at 120 hpf. Mean phasic total distance moved showed significance of phase [(F_4_,1940) = 549.23, *p* < 0.05] and treatment [(F_6_,485) = 2.56, *p* < 0.05], but not the interaction of treatment and phase [(F_24_,1940) = 0.90, *p* = 0.6060, and η_p_^2^ = 0.531] (Figure 1A). Phasic velocity showed significance of phase [(F_4_,1940) = 641.52, *p* < 0.05] and treatment [(F_6_,485) = 2.46, *p* < 0.05], but not the interaction of treatment and phase [(F_24_, 1940) = 0.86, *p* = 0.6535, and η_p_^2^ = 0.569] (Figure 1B). Time spent moving also showed significance of phase [(F_4_,1940) = 482.82, *p* < 0.05] and treatment [(F_6_,485) = 3.53, *p* < 0.05], but not the interaction of treatment and phase [(F_24_,1940) = 0.96, *p* = 0.5118, and η_p_^2^ = 0.500] (Figure 1C). Specifically, the 10 ppb As group exhibited hyperactivity in all dark phases for all three outcomes (*p* < 0.05) (Figure 1), while the 10 ppb Pb group had increased distance traveled and velocity only in the first dark phase (*p* < 0.05) (Figure 1). The 10 ppb mix group also was hyperactive in the first dark phase but was observed for all three outcomes similar to the 10 ppb As group (*p* < 0.05) (Figure 1). Furthermore, for time spent moving, the 10 ppb mix group was also hyperactive in dark phases 2 and 3, again the same as the 10 ppb As group (*p* < 0.05) (Figure 1C). The same observations were observed for the 100 ppb As and 100 ppb mix groups with hyperactivity in time spent moving in the second and third dark phases (*p* < 0.05) (Figure 1C). No significant alterations in behavior were observed for the 100 ppb Pb treatment group in any of the outcomes (*p* > 0.05) (Figure 1).

### 3.2. Morphological Measurements

**Figure 2:**
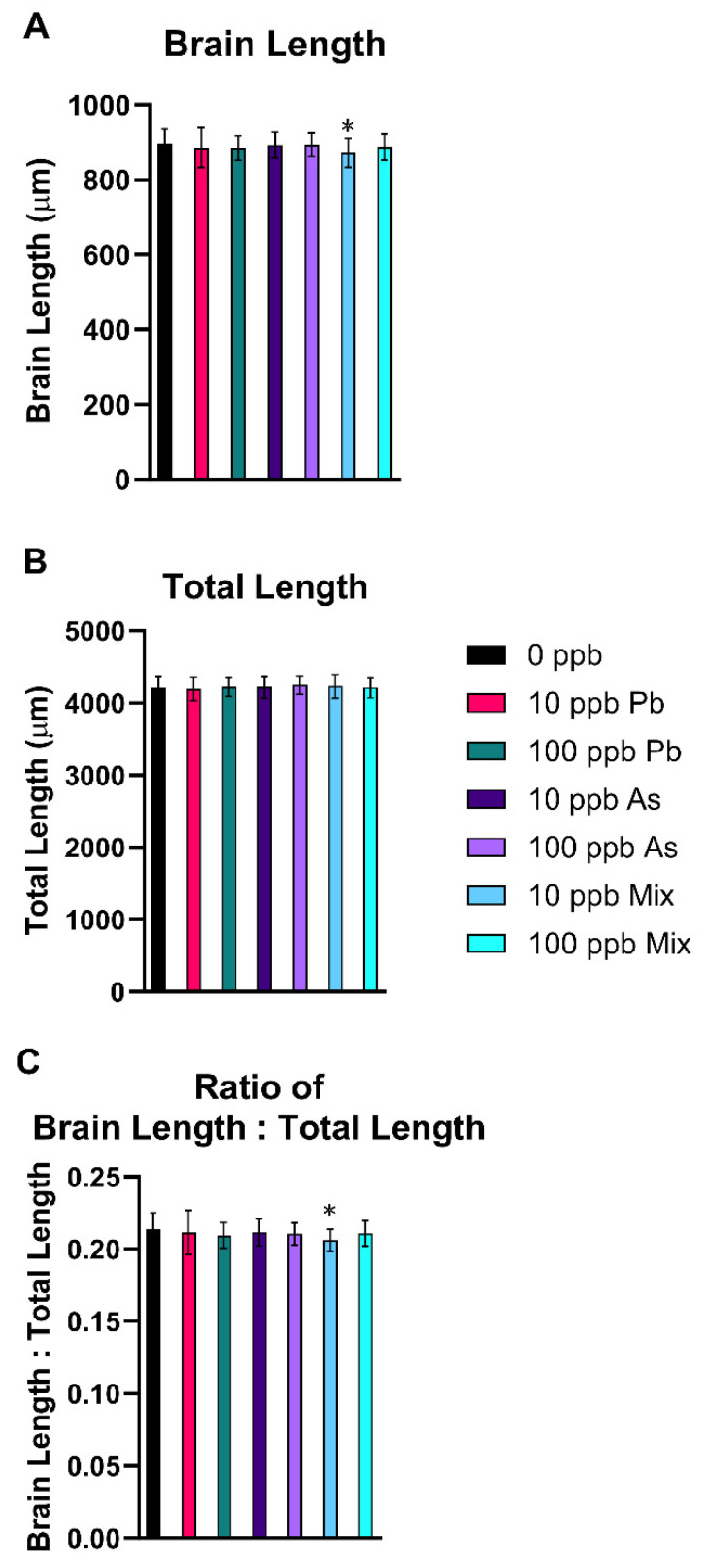
Alterations in morphology after developmental exposure to As, Pb, or a mixture of both from 1 to 120 hpf. A decrease in brain length in the 10 ppb mix treatment group was observed (**A**). No differences in total length were measured (**B**), but a decreased ratio of brain length to total length in the 10 ppb mix treatment group was also seen (**C**). n = 7 with 10 subsamples per treatment per replicate to total 70 fish per treatment group. Error bars represent standard deviation. * *p* < 0.05. Different letters indicate different statistical groupings in (**A**).

There was a significant decrease in brain length (*p* < 0.05) (Figure 2A) and the ratio of brain length to total larval length in the 10 ppb mix group (*p* < 0.05) (Figure 2C), indicating brain size was smaller than expected based on total length of the fish. There were no significant changes in total body length (Figure 2B), head length, head width, head length to total length ratio, or head width to total length ratio in any of the treatment groups (*p* > 0.05) (Table S2). 

### 3.3. Cerebral Vascular Measurements 

**Figure 3:**
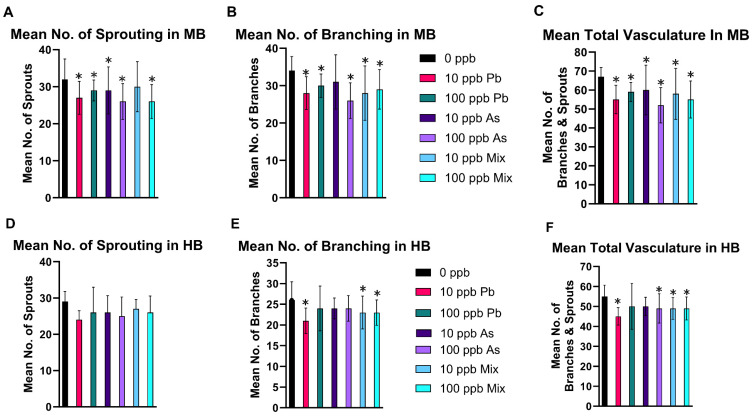
Decreases in mesencephalon (midbrain, MB) and cerebellum (hindbrain, HB) cerebral vasculature at 72 hpf after embryonic exposure to As, Pb, or a mixture of As and Pb. To evaluate cerebral vasculature development, several endpoints were assessed in the mesencephalon (midbrain, MB) including the number of sprouting blood vessels (**A**), the number of branches (**B**), and total vasculature (**C**). Similar measurements were ascertained in the cerebellum (hindbrain, HB) including number of sprouting blood vessels (**D**), the number of branches (**E**), and total vasculature (**F**). n = 5 with 10 subsamples per treatment per replicate for a total of 50 fish analyzed per treatment group. Error bars represent standard deviation. * *p* < 0.05.

**Figure 4:**
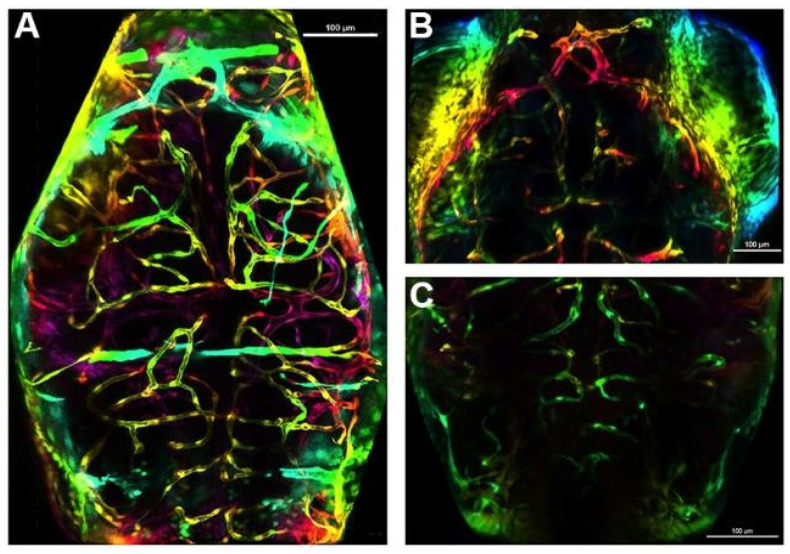
Examples of decreases observed in the mesencephalon (midbrain) and cerebellum (hindbrain) vasculature at 72 hpf. A representative brain of a fish in the control treatment group (**A**), of the mesencephalon of a fish in the 100 ppb As treatment group (**B**), and of the cerebellum of a fish in the 10 ppb Pb treatment group (**C**). Scale bar is 100 µM.

In the mesencephalon (midbrain), the number of sprouting pre-tip blood vessels decreased in the 10 ppb As and Pb groups (Figure 3A), in the 100 ppb As (Figure 4B) and Pb groups (Figure 3A), and in the 100 ppb mix group (Figure 3A) (*p* < 0.05). The mean number of connecting arteries (branches) decreased in all treatment groups, except in the 10 ppb As group (Figure 3B) (*p* < 0.05). Overall, a decrease in total vasculature (number of sprouts and branches) was seen in all treatment groups (Figure 3C) (*p* < 0.05), while the ratio of sprouting to branches was not changed (*p* > 0.05), indicating a similar reduction was occurring in the number of sprouts and branches (Table S3). Additionally, the length of the mesencephalon was decreased in the 10 ppb As, 10 ppb mix, 100 ppb Pb, and 100 ppb As groups (*p* < 0.05), but not in the 10 ppb Pb or 100 ppb mix groups (Table S3). Alternatively, the ratio of the number of branches to mesencephalon length was altered for these two treatment groups (10 ppb Pb and 100 ppb mix groups) (*p* < 0.05) (Table S3), signifying that impacts to vasculature were greater compared to length of the mesencephalon.

In the cerebellum (hindbrain), there was no significant impact on the number of sprouting blood vessels (*p* > 0.05) (Figure 3D), but a decrease in the number of central arteries (branching) occurred in the 10 ppb Pb, 10 ppb mix, and 100 ppb mix groups (*p* < 0.05) (Figure 3E). Total vascularization in the cerebellum was decreased in the 10 ppb Pb (Figure 4C), 10 ppb mix, 100 ppb As, and 100 ppb mix groups (*p* < 0.05) (Figure 3F), while the ratio of sprouting to branches was not significantly different (*p* > 0.05) (Table S3). The length of the basilar artery was significantly decreased in all groups (*p* < 0.05), except the 10 ppb Pb group (Table S3). The ratio of number of branches to basilar artery length was not changed (*p* > 0.05) (Table S3), indicating impacts to decreased branching were similar as decreases to the basilar artery length.

### 3.4. Gene Expression Alterations 

**Figure 5:**
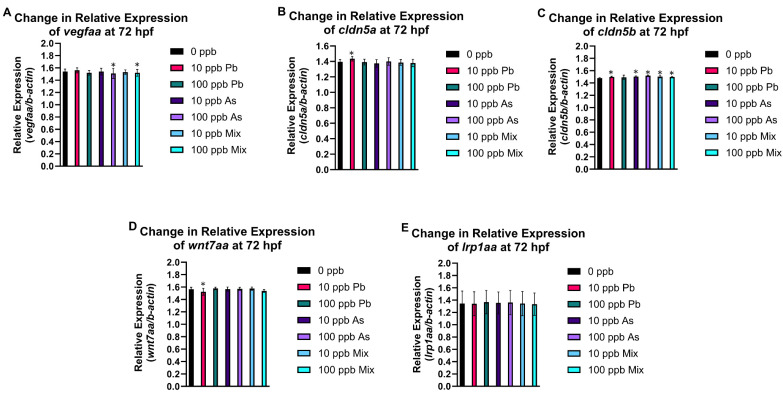
Expression of cerebral vasculature developmental and functional genes at 72 hpf after embryonic exposure (1–72 hpf) to As, Pb, or a mixture of As and Pb. There was a significant decrease in relative expression of *vegfaa* in 100 ppb As and 100 ppb mix treatment groups (**A**). A significant increase in *cldn5a* (**B**) and *wnt7aa* (**D**) expression occurred in the 10 ppb Pb treatment group. All treatment groups had an increase in *cldn5b* expression (**C**). There was no change in expression of *lrp1aa* for any treatment group (**E**). n = 6 (pools of 48–50 fish from each treatment in each biological replicate). Error bars represent standard deviation, * *p* < 0.05.

Expression of both claudins (*cldn5a* and *cldn5b*) increased in the 10 ppb Pb group (Figure 5B,C), while *wnt7aa* expression decreased (Figure 5D). Increased expression of *cldn5b* was also observed in the 10 ppb As and 10 ppb mix groups (Figure 5C). This was the only gene changed in these three treatment groups. The 100 ppb As and 100 ppb mix groups had decreased expression of *vegfaa* and increased *cldn5b* expression (Figure 5A,C). No significant changes in expression of *lrp1aa* were observed in any treatment group (Figure 5E). Results of all outcomes are integrated in Table S4, permitting comparison and relationships with gene expression alterations to be observed. 

## 4. Discussion

Pb exposure accounted for 62.5% of the global burden of developmental intellectual disabilities with main environmental exposure sources from the ingestion of water from leaded pipes, lead-contaminated dust, food from lead-glazed or lead-soldered containers, and from hand-to-mouth behavior of younger children (e.g., ingestion of lead paint chips) [14]. Global efforts were successful in eliminating leaded gasoline sales for motor vehicles, but controls and regulations for lead paint is only found in 48% of countries today. In the US, while recent numbers are declining, still over half a million children (aged 1–5 years) tested are over the blood lead action level, and as a known developmental neurotoxicant, these blood lead levels are still considered unacceptable [14]. 

Similar to Pb, As exposure across the globe is a major environmental health concern, with over 140 million people exposed to drinking water containing more than 10 ppb [13]. In addition to ingestion of contaminated drinking water, As exposure in the general population can also occur from contaminated food products. Unlike Pb, As contamination occurs from both natural and anthropogenic sources given the high presence of inorganic As in the groundwater of several countries across the globe. During early development, As can also cross into the brain via the blood stream and cause neurotoxic effects [63,64,65,66,67,68,69,70]. Both As and Pb induce oxidative stress, which in the brain changes the permeability of the BBB and alters brain morphology, brain edema, and hemorrhage [63,64,65,66,67,68,69,70]. With increasing recognition of the similar assumed mechanisms and targets of toxicity, it is increasingly recognized that studying the potential mixture effects of As and Pb is imperative. The present study was carried out to determine whether developmental Pb and As exposure is sufficient to induce neurotoxicity, including changes in brain vasculature using the developing zebrafish as a model for human health. Exposure periods in this study using the zebrafish align with in utero human development. 

The visual motor response assay assessed impacts of the As and Pb mixtures compared to the single metal exposures on behavior. Typical behavior in this assay is observed as decreased activity during light phases and a return to increased movement in dark phases. The most prominent response was hyperactivity in the 10 ppb Pb, 10 ppb As, and 10 ppb mix groups in all three outcomes in various dark phases. In addition, hyperactivity was also seen in the 100 ppb As and 100 ppb mix groups in dark phases of time spent moving. This finding is similar to multiple epidemiological studies reporting an association with Pb exposure and onset of attention deficit hyper disorder (ADHD) in children [71,72,73]. The volume of epidemiology studies evaluating As’s role in the development of ADHD is limited but growing. For example, As developmental exposure was linked to significant changes in typical ADHD battery tests used to score children and adults to measure their attention function, such as the simple reaction time test, continuous performance test, and selective attention test [74,75,76,77].

Moreover, a few studies investigated similar behavioral patterns in developing zebrafish with Pb or As exposures, but are overall inconsistent in their findings [48,52,78,79,80,81,82]. Zebrafish embryos exposed to 100 or 1000 ppb Pb had an increase in spontaneous movement from 20 to 30 hpf. This study also evaluated larval behavior at 120 hpf (25, 50, or 100 ppb Pb exposures), but reported hypoactivity in velocity in 50 and 100 ppb Pb groups [52]. Another developmental Pb neurotoxicity study also saw decreased responsiveness in a dose-dependent manner up to 6.21 ppb Pb in larvae exposed post hatch and evaluated at 168 hpf [78]. Similarly, at higher Pb exposure concentrations (1000 and 42,000 ppb), hypoactivity was reported, but these concentrations also cause serious malformations to the zebrafish’s swim bladder and tail, which influences lack of activity [83,84]. These findings are different than what was observed in our study with hyperactivity in the 10 ppb Pb treatment group and no alterations for the 100 ppb Pb treatment group, but may be explained by differences in exposure concentrations, duration of exposure, age at time of test, and behavior testing protocols. On the other hand, similar to our study, hyperactivity was reported in a study at concentrations between 3 and 94 ppb with locomotor activity increased [85]. 

Similar to human studies, there is also a lesser number of rodent and zebrafish studies evaluating impacts of a developmental As exposure on behavior compared to Pb [86,87,88]. For example, in the zebrafish photomotor response assay, increased activity was reported in the 18,750 to 37,500 ppb As^3+^ groups, but hypoactivity in the 56,250 to 75,000 ppb [46] groups was seen. These concentrations are multiple magnitudes above what was used in this study and used a different behavior test, making it difficult to compare the studies. Moreover, in our previous study [54] the As 120 hpf-LC_25_ was 40,180 ppb, the 120 hpf-LC_50_ was 55,420 ppb, and complete mortality was observed at 77,320 ppb and above at 120 hpf. As such, concentrations at which impacts to behavior were observed also were likely to have higher to near complete lethality in their study, meaning activity was not primarily related to neurotoxicity. 

Next, morphology measurements were completed to determine impacts on development and growth in comparison to past studies in rodents and zebrafish [48,52,78,82,89,90,91,92,93]. A decrease in brain length and change in the ratio of brain length to total length in the 10 ppb mix group were the only differences. This finding is consistent with other developmental Pb zebrafish studies reporting no changes in comparable measurements at similar exposure concentrations [78,82]. 

For investigating the potential impacts of the As and Pb exposures on the developing cerebral vascular system, a transgenic zebrafish model in which all blood vessels fluoresce was incorporated. This emphasis was chosen based on observations of microbleeds occurring in the brain of developing zebrafish in a previous study at higher exposure concentrations [54]. The vasculature network is made of an extensive number and variety of blood vessels, all of which ensure that efficient blood flow occurs to all tissues to deliver oxygen and nutrients. In zebrafish, central arteries begin to penetrate the telencephalon, mesencephalon, and cerebellum directly from the basal communicating artery and posterior communicating segments and from the basilar artery. These central arteries make connections to and begin to feed existing venous vessels on the brain surface. This network of arteries and veins provides the brain tissue with nutrients needed for development of and proper function of the brain tissue. Accurate patterning and development of vessels are important to maintain cerebral function. At 24 hpf, the heartbeat begins and the perineural vessels migrate and invade brain tissue, forming the primordial cerebellum channels (PHBC) and the basilar artery (BA). At 60 hpf, development of major brain vasculature is completed by vasculogenesis as indicated by the presence of complete pairs of anterior mesencephalic central artery (AMCtA), prosencephalic artery (PrA), and communicating vessel (CMV) [94]. Lastly, by 72 hpf, the zebrafish’s blood–brain barrier is formed and the vascular structure including anterior mesencephalic central artery (AMCtA) and central arteries (CtAs) exhibits restrictive barrier properties [95], but the selectivity and permeability of the zebrafish BBB for the exposure period of this study (1–72 hpf), only restricts molecules greater than approximately 961 Da [95,96]. In addition, given past studies show that molecules smaller than sodium fluorescein (376 Da) can cross the BBB at 48 hpf, it is a reasonable assumption that Pb and As, even after the formation of a BBB, could cross the BBB [97,98,99] as the size of a Pb atom is approximately 202 Da and the size of a As is approximately 75 Da. 

Several genes were included to link alterations in cerebral vasculature with molecular alterations including those related to angiogenesis and vasculogenesis (i.e., *vegfaa*, *wnt7aa*, and *lrp1aa*) and genes associated with tight junctions (i.e., *cldn5a* and *cldn5b*). Endothelial cells lining the intracerebral vessels in zebrafish immediately express specific genes for barriergenesis and angiogenesis and for molecular transport to acquire restrictive membrane properties as they migrate [100]. Capillary brain endothelial cells, as part of the BBB, specialize and increase in barrier tightness using proteins such as some claudins to form additional cell–cell junctions via epithelial apical junctions (AJC). The brain endothelium is sealed by other proteins, including claudins that form tight junctions to prevent paracellular diffusion of toxicants. Claudin-5 has the highest expression in the brain blood vessels among the entire family of claudins. *cldn5a* and *cldn5b* are the zebrafish paralogs. cldn5a expression has an essential role in ventricular lumen expansion before the establishment of an embryonic BBB [101] and formation of the neuroepithelial barrier. *cldn5a* is expressed in the brain vasculature starting at 48 hpf [101,102,103] with mRNA expression of *cldn5a* visualized at 72 hpf and onwards. During the development of the BBB cldn5a contributes to the formation of TJs in between endothelial cells. During hypoxic conditions in mouse brain microvascular endothelial cells, Cldn5 localizes inside these cells, migrating from the TJ formed between two endothelial cells [104]. This re-localization of Cldn5 also occurs in brain microvascular endothelial cells collected from current stroke patients and in zebrafish. The redistribution of cldn5a leads to increased permeability, which is partially decreased when autophagy capacity restored and aggregated cldn5a is degrade [41]. At 24 hpf, *cldn5b* is visible, as is its restriction to vasculature. From 48 to 72 hpf, *cldn5b* is expressed in brain blood vessels, brain vasculature, the cardiovascular system, cranial blood vessel endothelial cell, and blood vessel endothelium bicellular tight junction [102,105,106,107]. By 72 hpf, zebrafish larvae express cldn5b in the MCeV, PrA, and ACeV. These main arteries and veins support delivery of nutrients, developmental proteins, and functional proteins. At 72 hpf, all treatment groups had increased expression of *cldn5b*, while increased expression of *cldn5a* was only observed in the 10 ppb Pb group, which agreed with alterations in this gene by Pb in another part of this study. This increase in *cldn5b* could be in response to disruption of the total vasculature system observed in all treatment groups in the mesencephalon and the need to increase or repair the barrier as observed. Moreover, throughout all outcomes, the 10 ppb Pb group showed a unique pattern, which aligns with the only treatment group with changes in *cldn5a*. Alternatively, given *cldn5b* is expressed earlier in development, if expression was assessed at a slightly later developmental age, alterations in this gene may also be observed (i.e., 10 ppb Pb is most sensitive to detect this change early). 

At 20 hpf, the PHBC are formed along the anterior–posterior axis in the ventral side of the hindbrain [94]. Angiogenic sprouts begin to surface from the PHBC laterally and perpendicularly (dorsally) from 28 to 32 hpf. The invasion of central arteries in the hindbrain occurs around 32 hpf. In the first 24 h, *wnt7aa* is expressed in the hindbrain. Subsequently, *wnt7aa* is expressed in the cerebellum and in the dorsal midbrain. *wnt7aa* is one of four wnt7 genes associated with the Wnt ligand-specific signaling pathways that regulate facets of neuronal cell differentiation. In the brain endothelial cells, *gpr124* and *reck* enable a selective response to *wnt7a/wnt7b* paralogs. This response is frizzled signaling leading to an increase in wnt7 orthologs for development of central arteries in specific regions in the brain, including the hindbrain [108,109]. At 72 hpf, *wnt7aa* expression only decreased in the 10 ppb Pb group. The decrease in CtAs (branches) in the cerebellum of zebrafish in the 10 ppb Pb group agrees with the changes in transcription. While a decrease in branching was also observed in the cerebellum in the 10 and 100 ppb mix groups, the perturbations were more drastic in the 10 ppb Pb group. There are a few studies that report that developmental Pb exposure in rats leads to decreased Wnt7a protein expression [110], similar to as observed in our study. 

The 10 and 100 ppb As groups (and 10 and 100 ppb mix groups) had decreased time spent moving in the last two dark phases in the visual motor response assay, but the 100 ppb As group had more prominent perturbations in additional outcomes that were not observed in the 10 ppb As group (e.g., number of branches in mesencephalon, decreased total vasculature in cerebellum, and decreased expression of *vegfaa*). In zebrafish, *vegfaa* acts upstream of circulatory system development and regulates vasculature development [111]. From 1 to 72 hpf, the zebrafish brain is vascularized by sprouting angiogenesis, which strictly depends on *vegfaa* signaling [112,113,114]. Moreover, excess production of *vegfaa* in neuronal tissue results in malformation of brain blood vessels, indicating the importance of balanced production of *vegfaa* for brain blood vessel development and maintenance in zebrafish. In addition, a TALEN-generated zebrafish *vegfaa* mutant was developed to further study the role of *vegfaa* in artery and vein formation and displayed loss of mesencephalic veins (MsVs) and central arteries (CtAs), which are both a product of sprouting angiogenesis [111,115,116]. Staining also showed *vegfaa* is expressed at the midbrain–hindbrain boundary (MHB) and the midbrain region at 24 hpf. A principal mechanism of angiogenic growth in the brain occurs via the onset of sprouting angiogenesis. Sprouting angiogenesis is the extension of blood vessels, which includes specialization of endothelial cells leading to tip cells formation, production, and relocation of specific endothelial cells, and cell adhesion. The mechanism that leads to sprouting angiogenesis and intersegmental blood vessel growth was explored and detailed in zebrafish [117,118]. The decrease in *vegfaa* expression in the 100 ppb As and 100 ppb mix groups overall aligns with other perturbations observed, including decreased sprouting, number of branches, and total vasculature in the mesencephalon. 

The last gene evaluated in this study, *lrp1aa*, is the ortholog of human LRP1 (LDL receptor related protein 1) and acts upstream of or within the regulation of vasculature development [119]. It is located in the membrane and is a required protein for the alpha 2-macroglobulin-mediated clearance of secreted amyloid precursor protein (APP) and beta-amyloid [120,121]. *lrp1aa* was a gene of interest as APP in human neurons, and *appa* and *appb* orthologs in zebrafish are impacted by exposure to As or Pb and changes in expression of APP and its orthologs in zebrafish and rodent models are proven to be related to changes in the development and maintenance of blood vessels, especially in the brain [122]. However, there were no significant changes in lrp1aa expression at 72 hpf. 

Overall, this study found that the most prominent impacts to behavior occurred in the 10 ppb As, Pb, and mix groups, but that hyperactivity in time spent moving was also observed for the 100 ppb As and mix treatment groups, which overall is concordant with epidemiological studies reporting increases in ADHD. The behavior impacts the 10 ppb groups, aligned with the 10 ppb mix treatment, being the only one in which a decrease in brain length occurred, but when evaluating more detailed measurements on the cerebral vascular system, all treatment groups were impacted. In addition, the mesencephalon had increased perturbations among the treatment groups compared to the cerebellum for the outcomes assessed. Among all the outcomes, a general pattern of the 10 ppb Pb group driving the 10 ppb mix group alterations and the 100 ppb As group aligning with the 100 ppb mix group changes emerged. In addition, the 10 ppb Pb group exhibited a unique pattern compared to the other treatment groups among multiple outcomes, further supporting the sensitivity and non-linear concentration response that was observed in other studies at lower Pb exposures [123,124]. In conclusion, this study built on prior studies of each of these two compounds individually to add new findings specific to cerebral angiogenesis and vasculogenesis when considering mixture toxicity. While As and Pb are two common global environmental contaminants, it is recognized that there are likely other chemicals that may also be present in mixtures in which the general population is exposed. Further work is needed to continue to address developmental neurotoxicity risks of more complex chemical mixtures. 

## Data Availability

Data is available upon request.

## Supplementary Materials

The following supporting information can be downloaded at: https://www.mdpi.com/article/10.3390/toxics12090624/s1, Supplementary Methods; Figure S1: micro-bleeds observed during acute mixture at 96 h post fertilization (hpf); Figure S2: cerebral vasculature of fli:1a (EGFP) transgenic fish at 72 h post fertilization (hpf); Table S1: primer sequences used in qPCR analysis; Table S2: measurements with no statistical changes for morphology; Table S3: mesencephalon and cerebellum cerebral vascular measurements; and Table S4. Summary of results from all endpoints evaluated in this study.
