# Supplementary material for: Cerebral Vascular Toxicity after Developmental Exposure to Arsenic (As) and Lead (Pb) Mixtures
Source: Toxics. 2024 Aug 24;12(9):624. doi: 10.3390/toxics12090624 (PMC11435665; doi:10.3390/toxics12090624)
Supplement: Supplementary file 1 [file toxics-12-00624-s001.zip › toxics-3105244-supplementary.pdf]

SUPPLEMENTARY MATERIALS

Cerebral vascular toxicity after developmental exposure to arsenic (As) and lead (Pb) mixtures

Keturah Kiper[a], Breeann Mild[a], Jenny Chen[a], Chongli Yuan[b], Ellen M. Wells[a], Wei Zheng[a], and Jennifer L. Freeman[a]*

[a]School of Health Sciences, Purdue University, West Lafayette, IN, USA

[b]Davidson School of Chemical Engineering, Purdue University, West Lafayette, IN USA

- Supplementary Methods. Confocal microscopy measurements of cerebral vascular development
- Figure S1. Micro-bleeds observed during acute mixture at 96 hours post fertilization (hpf).
- Figure S2. Cerebral vasculature of fli:1a (EGFP) transgenic fish at 72 hours post fertilization (hpf).
- Table S1. Primer sequences used in qPCR analysis.
- Table S2. Measurements with no statistical changes for morphology.
- Table S3. Mesencephalon and cerebellum cerebral vascular measurements.
- Table S4. Summary of results from all endpoints evaluated in this study.

Supplementary Methods. Confocal microscopy measurements of cerebral vascular development

NIS-Elements imaging software was used to capture images with the parameters set as: galvano, step size of 2 µm, pinhole: 1.2 AU, size: 1024, control by: 1 frame/ second, FITC laser. The average number of stacks for these images was 325. Using 2D and volume view, the cerebral vasculature in the mesencephalon (midbrain) and cerebellum (hindbrain) were evaluated. The presence of the three pairs of arteries connected to the basal communicating artery (BCA) and the basal artery (BA) in each zebrafish was noted as a sign that development of major vasculature had occurred. The number of sprouting angiogenic tips connecting mesencephalic central arteries (MCtAs) from the choroid vascular plexus (CVP) to the BCA and the number MCtAs connecting to the BCA in the mesencephalon were recorded (as branches). A complete MCtA was defined as one that connects to the CVP and the BCA via three important pairs of connecting MCtAs: the anterior mesencephalic central artery (AMCtA), communicating vessel (CMV), and prosencephalic artery (PrA). In the cerebellum, the number of sprouting CtAs from the dorsal wall of each primordial hindbrain channel (PHBC) and the number of pathfinding CtA branches that progressed to lumen formation (i.e., connection of CtAs to BA) were measured; a complete CtA will sprout from the PHBC and connect to the BA. Next the distance from the BCA to the telencephalon (midbrain) and length of the BA (hindbrain) were determined. These lengths were used to account for overall changes in the size of the brain of each treatment group. The confocal z-stacks were converted to a 3D image using NIS-Elements analysis software, binarized to establish a threshold, and objects (i.e., MCtAs, CtAs, PHBC, BA, BCA, and CVP) identified to calculate the surface area and volume of cerebral blood vessels present.

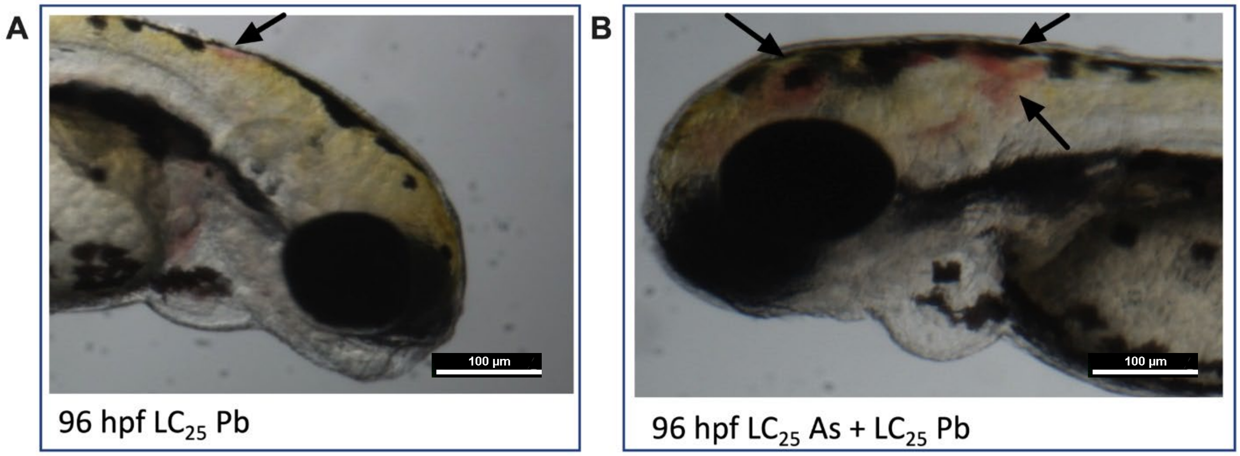

Figure S1. **Micro-bleeds observed during acute mixture at 96 hours post fertilization (hpf).** After 96 hours of exposure to 39 ppm (mg/L) Pb (A) or a mixture of 39 ppm Pb and 40.18 ppm As (B) micro-bleeds were observed in the hindbrain and midbrain in live zebrafish larvae indicating perturbations to the cerebral vascular system (black arrows). Scale bars are 100 µM.

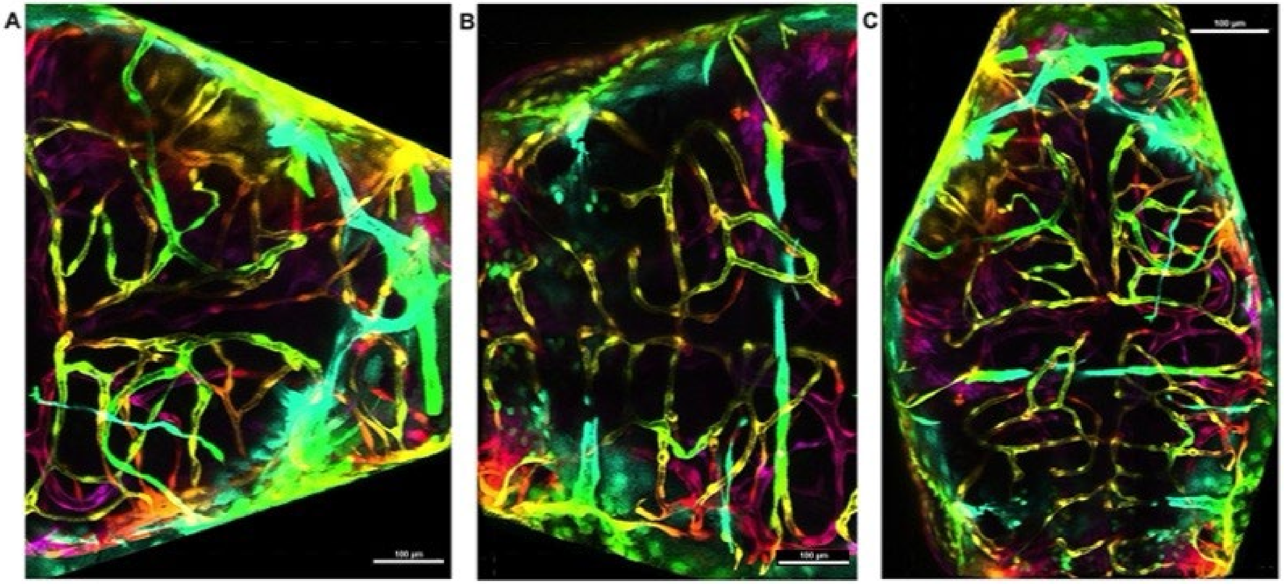

Figure S2. Cerebral vasculature of fli:1a (EGFP) transgenic fish at 72 hours post fertilization (hpf). Mesencephalon (A), cerebellum (B), and total brain (C) in a control treatment group. Different colors represent structures on different planes. Scale bars are 100 μM.

Table S1. Primer sequences used in qPCR analysis.

Target Gene	Human Ortholog	Ref Seq	Primer Sequence (5'–3')	Function[a]
cldn5a	*CLDN5* (claudin 5)	RefSeq:NM_213274	F: GTCAAATACGCGCCCACTAAAA<br>R: CACACACACACACACACTCAAG	Involved in establishment of endothelial barrier; localizes to apical junction complex; expressed in CNS.
cldn5b	*CLDN5* (claudin 5)	RefSeq:NM_001006044	F: GACTGTCATCTCTGCGGTTTTG<br>R: TGGATTTGATGGACTCCGTGTT	Localizes to bicellular tight junction; integral component of membrane and plasma membrane; expressed in cardiovascular system, retina, and ventral mesoderm.
lrp1aa	*LRP1* (LDL receptor related protein 1)	RefSeq:XM_021479679	F: GATTACGACGGATACCTGCTGT<br>R: AGGGTTTGATAGGAGCGTTGAG	Involved in regulation of vasculature development; expressed in blood vessel and caudal vein plexus.
vegfaa	*VEGFA* (vascular endothelial growth factor A)	RefSeq:NM_131408	F: AGAGCTGCTGGTAGACATCATC<br>R: CATTCGAGCGCCTCATCATTAC	Exhibits growth factor activity; involved in several processes, including circulatory development and vasculogenesis.
wnt7aa	*WNT7A* (Wnt family member 7a)	RefSeq:NM_001025540	F: TGGATGTGACAAGGAGAAGCAA<br>R: GCTCAGACCGTAGCGAATATCA	Frizzled binding activity; involved in Wnt signaling pathway, cell fate commitment, and neuron differentiation.
actb2	*ACTB* (actin beta)	RefSeq:NM_181601	F: CTAAAAACTGGAACGGTGAAGG<br>R: AGGCAAATAAGTTTCGGAACAA	Structural constituent of postsynaptic actin cytoskeleton; predicted to be involved in synaptic vesicle endocytosis; included as reference gene.

[a]Biological function as defined as zfin.org

Table S2. Measurements with no statistical changes for morphology.

Treatment	Head Length (μm)	Head Width (μm)	Head Length/ Total Length	Head Width/ Total Length
0 ppb	752.63 ± 42.62	639.98 ± 21.80	0.1785 ± 0.110	0.1519 ± 0.005
10 ppb Pb	752.37 ± 40.80	641.88 ± 37.20	0.1793 ± 0.008	0.1530 ± 0.009
100 ppb Pb	753.96 ± 41.95	641.12 ± 22.92	0.1784 ± 0.008	0.1517 ± 0.005
10 ppb As	757.27 ± 36.60	640.48 ± 20.48	0.1785 ± 0.009	0.1518 ± 0.005
100 ppb As	753.96 ± 45.53	642.86 ± 23.05	0.1775 ± 0.009	0.1514 ± 0.006
10 ppb Mix	753.00 ± 40.68	636.99 ± 30.29	0.1779 ± 0.007	0.1505 ± 0.006
100 ppb Mix	747.47 ± 42.76	634.72 ± 21.26	0.1774 ± 0.009	0.1507 ± 0.005

Table S3. Mesencephalon and cerebellum cerebral vascular measurements.

Treatment	Mesen-cephalon Length (μM)[a]	Mesen-cephalon No. of branches to length ratio	Mesen-cephalon sprouting to branch ratio	Basilar Artery Length (μM)	Cerebellum No. of branches to length ratio	Cerebellum sprouting to branch ratio
0 ppb	158.82 ± 31.67	0.2133 ± 0.06	0.2292 ± 0.14	140.63 ± 20.50	0.1917 ± 0.05	1.14 ± 0.21
10 ppb Pb	157.72 ± 31.70	0.1731 ± 0.04*	0.1831 ± 0.20	128.17 ± 23.82	0.1723 ± 0.04	1.15 ± 0.19
100 ppb Pb	135.72 ± 22.98*	0.2177 ± 0.05	0.2292 ± 0.13	121.73 ± 16.53*	0.2031 ± 0.05	1.05 ± 0.21
10 ppb As	141.81 ± 28.00*	0.2091 ± 0.04	0.2173 ± 0.16	119.38±16.46 *	0.2055 ± 0.02	1.09 ± 0.26
100 ppb As	129.5 ± 26.97*	0.2155 ± 0.05	0.2055 ± 0.11	117.1 ± 20.41*	0.2091 ± 0.04	1.09 ± 0.18
10 ppb Mix	138.84 ± 27.50*	0.2182 ± 0.06	0.2073 ± 0.15	120.52 ± 21.4*	0.1927 ± 0.04	1.19 ± 0.17
100 ppb Mix	142.92 ± 26.61	0.1815 ± 0.04 *	0.2069 ± 0.16	124.4 ± 24.73*	0.1946 ± 0.04	1.12 ± 0.24

[a]Mean ± standard deviation

*p<0.05. No significant changes for mesencephalon or basilar artery sprouting to branch ratio or for cerebellum number of branches to length ratio.

Table S4. Summary of results from all endpoints evaluated in this study.[a,b]

Treatment	Behavior			Morphology				Cerebral vascular morphology												qPCR				
	TD	V	TSM	BL	HL	HW	TL	CBS	CBB	CB TV	BAL	CB B:L	CB S:B	MBS	MBB	MB TV	MBL	MB B:L	MB S:B	*cldn5a*	*cldn5b*	*lrp1aa*	*vegfaa*	*wnt7aa*
**10 ppb Pb**	blue	blue	blue						red	red				red	red	red		red		blue	blue			red
**100 ppb Pb**											red			red	red	red	red							
**10 ppb As**	blue	blue	blue								red			red		red	red				blue			
**100 ppb As**			blue							red	red			red	red	red	red				blue		red	
**10 ppb Mix**	blue	blue	blue	red					red	red	red				red	red	red				blue			
**100 ppb Mix**			blue						red	red	red			red	red	red		red			blue		red	

[a]Abbreviations: TD = Total distance; V = Velocity; TSM = Time spent moving; BL = Brain length; HL = Head length; HW = Head width; TL = Total length; MBS = mesencephalon brain sprouts; MBB = mesencephalon branches; MB TV = mesencephalon total vasculature; MBL = mesencephalon length; MB B:L = mesencephalon branches to length; MB S:B = mesencephalon sprouting to branch ratio; CBS = cerebellum sprouts; CBB = cerebellum branches; CB TV = cerebellum total vasculature; BAL = basilar artery length; CB B:L = cerebellum branches to length; CB S:B = cerebellum sprouting to branch ratio

[b]Color key: blue: significant increase; red: significant decrease
